# An Involuntary Proving of the Fruit Wasp

**Published:** 1894-12

**Authors:** C. F. Menninger

**Affiliations:** Topeka, Kansas; Topeka Homœopathic Academy of Medicine and Surgery


### AN INVOLUNTARY PROVING OF THE FRUIT WASP.

C. F. MENNINGER, M. D., TOPEKA, KANSAS.

(Topeka Homœopathic Academy of Medicine and Surgery.)

J. H. W., aged forty-eight, a tall, raw-boned, well-built man of bilious temperament, shortly after eating dinner (12 M.), and while at work, ate a part of an apple which had been stung by a common fruit wasp (or "yellow-jacket"). [*Vespa media.*—ED.]

He says that within a very short time afterward his mouth filled up with saliva, requiring him to spit a number of times. Thereupon followed nausea, and he immediately vomited up all of his dinner and the apple he had just eaten. Within a few moments after he felt an itching in the axillæ and then under the knees, in the popliteal spaces. From there it spread upward on his thighs, involving the genital organs and buttocks, and upward all over his abdomen and back. Simultaneously it spread from the axillæ upward over neck, face, and head, and downward over arms and hands. Next he noticed that his lips and tongue began to be affected with tingling, itching, and swelled sensation, and this extended into the throat, fauces, lungs, and stomach, until he seemed fairly afire, within and without. Great difficulty of respiration and of swallowing—the respiratory efforts being mostly affected. With the dyspnœa there came a cough, dry, harsh, and expectorationless, which seemed to proceed from this irritation low down in the trachea. He said the throat swelled up and the lips puckered up.

On walking to the office of his physician he felt a swimming of his head, a reeling, with a feeling of falling backward, which was all aggravated by walking or standing, but relieved by lying down. On entering the doctor's office, with expression of anxiety depicted in his face, which was a bluish scarlet, puffy, and eyes staring, he said, "Hurry up; I am dying with the hives."

The skin all over the body was scarlet, and raised in hive-like manner. It was hot to the touch, and caused him to scratch violently, saying he was all afire. There was marked œdema of the prepuce, of theupper eyelids (lower lids somewhat, but not so much), and of the lips.

One dose of Apis $^{cm}$ relieved him in fifteen minutes, when he broke out with a gentle moisture all over his body, although the room was not over 70° F., and he had not exercised for some thirty minutes prior.

Prior to this experience he had been apparently perfectly well; had worked at the stone-cutter's trade for some time, and had had no hives, nor any stomach disturbance at all.

Let us now take a brief review of the main symptoms of this involuntary proving, in the order in which they appeared, and devoid of the connecting narrative.

1. Nausea and vomiting, preceded by spitting.

2. Itching, burning, hive-like sensation, commencing in axillæ, under the knees, and thence all over the body. Skin first scarlet, fever-like, and, on scratching, urticaria-like elevations, with fire-like burning, followed by a deepening of the color almost to a purplish red.

3. Swelling of skin, œdema of face, eyelids, and lips, giving appearance of one who had just awakened out of prolonged sound sleep; genital organs swollen entirely out of shape; a contracted or full sensation of respiratory passages, with a puckering and numb sensation of lips, attended by a dry cough, from irritation in larynx and trachea, with dyspnœa, especially marked at inspiration.

4. Swimming, reeling, vertigo, with inclination to fall backwards, aggravated by standing and walking, and better while lying down.

5. Anxiety, with fear of death, due to the impeded or oppressed respiration and the excessive burning, like fire, within and without.

6. Relief coming on by gentle moisture over the entire body.

From the totality of these symptoms we were led to prescribe Apis-mel., of which but one single dose of the CM potency was given, the results of which have been given in the narrative.

Now, judging from this result and the entire picture of symptoms, we have a most vivid and exact demonstration of the law of similars: "A drug (Apis-mellifica) producing disturbances in a comparatively healthy body is capable of relieving or entirely obliterating similar (fruit wasp) disturbances when found in the sick person;" and thereby adding one more link in the chain of evidence demonstrating the universal application of this law; and, if universal, then a natural law; and thereby establishing the fact that Homœopathy is the *science* of therapeutics.

Let us now briefly note the sphere of action of this drug. Like all the poisons of the animal kingdom, its action is characterized by its violence, intensity, and rapidity, and by its tendency (which I feel would be demonstrated, if carefully proven) to alter both function and structure. By careful proving, animal poisons have all been found to produce symptoms similar to diseases which are never of asthenic character, but always of a destructive form, tending to local as well as to general death of the body; and, therefore, because of this profound action, are suitable to deep-seated diseases, as typhoid states and fevers, erysipelatous and phlegmonous inflammations, and those deep-seated disease conditions that underlie and modify acute disorders. From the *Hymenoptera* or membrane wings we have the *Apis-mellifica*, the honey-bee, the *Vespa vulgaris*, the wasp, the *Vespa crabo*, the hornet, the *Vespa media*, the yellow-jacket, the *Bombus* or bumble-bee, and the *Formica rufa*, one of the large family of ants (the red ant). The poisons from all these possess very similar pathological action, affecting the cellular tissues, producing œdema of the skin and mucous membrane. The skin is also covered with an eruption of a scarlet-fever type, or of urticaria-like elevations. The irritation of the skin passes on to the extent of inflammation from the simplest and mildest to the intensest erysipelatous destruction and gangrene.

In this case the irritation of the terminal filaments of the pneumogastric nerve, in the mucous membrane of the stomach, produced first the reflex excitation of the salivary glands, and then was followed by the direct irritation of the stomach, causing expulsion of the ingesta by vomiting. The bulk of the offending substance being removed (not soon enough, however, to prevent other symptoms), the patient was spared the most violent and baneful effects that would have followed had the entire effect of the drug been made manifest. Only a moment's peace was afforded him, however, when it made its appearance in the cellular tissues of the skin and mucous membrane. Congestion of these surfaces, followed by inflammation, account for all the scarlet-fever-like, urticaria-like skin appearances, the œdema, the dyspnœa, and cough.

Then the co-ordinating centres were attacked, and reflexly, through the dyspnœa and the intense fiery burning within and without, the mind—citadel of reason and consciousness—itself felt the effect.

Thus we trace the effect of this violent poison upon the prover, transpiring in less time than it is capable of being told. —*Medical Arena*, November, 1894.

